# Diagnosis of Conversion Disorder Using Diffusion Tensor Tractography and Transcranial Magnetic Stimulation in a Patient with Mild Traumatic Brain Injury

**DOI:** 10.3390/diagnostics9040155

**Published:** 2019-10-22

**Authors:** Sung Ho Jang, You Sung Seo

**Affiliations:** Department of Physical Medicine and Rehabilitation, College of Medicine, Yeungnam University 317-1, Daemyungdong, Namku, Taegu 705-717, Korea; rehab6467@hanmail.net

**Keywords:** mild traumatic brain injury, conversion disorder, diffusion tensor imaging, transcranial magnetic stimulation

## Abstract

We report on a patient with mild traumatic brain injury (TBI) who was diagnosed with conversion disorder for severe weakness of an arm, which was demonstrated using diffusion tensor tractography (DTT) and transcranial magnetic stimulation (TMS). A 23-year-old right-handed female suffered from head trauma resulting from a pedestrian car accident. She underwent rehabilitative management for memory impairment and central pain. At 14 months after onset, she complained of severe weakness of her right arm, which was detected in the morning after sleeping (right shoulder abductor: 3/5, elbow flexor: 3/5, wrist extensor: 1/5, finger flexor: 1/5, and finger extensor: 1/5). Electromyography study for peripheral neuropathy performed at 2 weeks after onset of weakness showed no abnormality. On a 14-month DTT configuration, the integrities of the left corticospinal tract (CST), supplementary motor area-corticofugal tract (SMA-CFT), and dorsal premotor cortex (dPMC)-CFT were well-preserved. Significant differences were not observed for the fractional anisotropy (FA), mean diffusivity (MD), and tract volume (TV) values of the CST, SMA-CFT, and dPMC-CFT in both hemispheres between the patient and ten right-handed age- and sex-matched normal subjects (*p* > 0.05). On a 14-month TMS study, MEPs obtained at the right abductor pollicis brevis muscle showed no abnormality. Using DTT and TMS, conversion disorder was demonstrated in a patient with mild TBI, who showed severe weakness of an arm. Our results suggest the usefulness of an evaluation of the CST and CFTs from the secondary motor areas using DTT, and the CST using TMS for patients who complain of motor weakness due to conversion disorder.

## 1. Introduction

Conversion disorder is a somatoform disorder defined by pseudoneurologic symptoms [1]. Correct diagnosis of conversion disorder presenting with motor symptoms has been limited by the lack of gold standard diagnostic tests and the absence of a universally accepted set of positive diagnostic criteria [1]. As a result, the diagnosis of conversion disorder presenting with motor weakness has been based on the physical examination such as the Hoover test [2,3].

For a correct diagnosis of conversion disorder with motor weakness, the possibility of injury of the neural tracts for motor execution, especially the corticospinal tract (CST), and the corticofugal tract (CFT) from the secondary motor area for motor planning, should be ruled out [4,5,6,7]. Diffusion tensor tractography (DTT) provides a three-dimensional evaluation of the integrity and pathway of the injured CST or CFTs from the secondary motor area, while transcranial magnetic stimulation (TMS) can distinguish between the CST and non-CST, and estimates the amount of the CST by analyzing the characteristics of the motor evoked potential (MEP) [8,9]. Therefore, we think the combined use of DTT for the CST and CFT, and TMS for the CST could be helpful in the precise diagnosis of conversion disorder with motor weakness.

Many studies have reported that the conversion disorder was diagnosed by demonstrating no abnormality of the CST using the characteristics of MEP on TMS in patients who complained of motor weakness [10,11,12,13,14,15]. However, no DTT-based study which confirmed the conversion disorder in patients with motor weakness has been reported so far, although a case study demonstrated the CST injury on DTT in a patient with quadriparesis who was misdiagnosed with the conversion disorder [16].

In this study, we report on a patient with mild traumatic brain injury (TBI) who was diagnosed with conversion disorder for severe weakness of an arm, which was demonstrated using DTT and TMS.

## 2. Case Report

**Figure 1:**
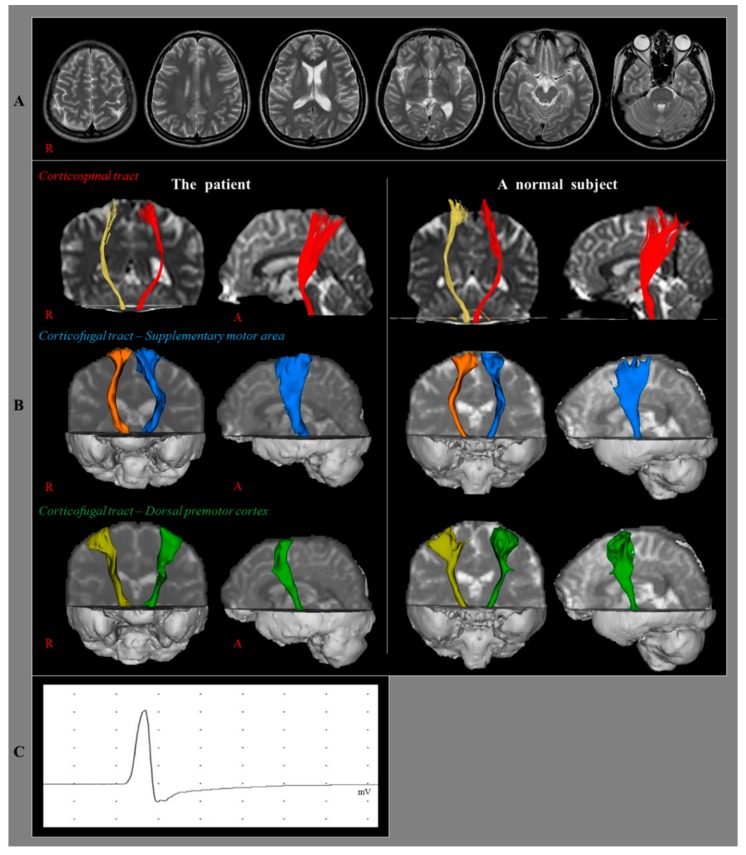
(**A**) Brain magnetic resonance imaging(MRI) images at 14 months after onset show no abnormal lesions. (**B**) Results of diffusion tensor tractography (DTT) for the corticospinal tract (CST) and corticofugal tract (CFT). On a 14-month DTT configuration, the left CST, CFT from supplementary motor area, and CFT from dorsal premotor cortex show similar configuration with a normal subject (24-year old female). (**C**) Results of transcranial magnetic stimulation (TMS). On a 14-month TMS study, motor evoked potential (MEP) is obtained at the right abductor pollicis brevis muscle during stimulation of the left hemisphere with 60% of maximal output (the MEP of shortest latency; 21.0 msec, amplitude: 5.1 mV).

A 23-year-old right-handed female and ten right-handed age- and sex-matched normal subjects (mean age 24.85 ± 4.32 years) without a history of neurological, physical, or psychiatric illness were included in this study. The patient suffered from head trauma resulting from a pedestrian car accident. While walking on a crosswalk, she was hit by a sedan from the side and then her head hit the ground while falling down. The patient experienced post-traumatic amnesia for approximately 10 min from the time of the accident without loss of consciousness. Her Glasgow Coma Scale score was 15. She underwent rehabilitative management for memory impairment and central pain from approximately four weeks after the onset of head trauma. At 14 months after onset, she complained of severe weakness of her right arm, which was detected on the morning after sleeping (right shoulder abductor: 3/5, elbow flexor: 3/5, wrist extensor: 1/5, finger flexor: 1/5, and finger extensor: 1/5). No specific lesion was observed on a brain MRI (T1-weighted, T2-weighted, and fluid-attenuated inversion recovery [FLAIR] images) (Figure 1A). Electromyography study for peripheral neuropathy performed at 2 weeks after the onset of weakness showed no abnormality. After approximately 4 months after the onset of weakness, the weakness of her right arm was recovered slowly and gradually to a nearly normal state and she was able to write a letter and use chopsticks. The research has obtained the patient’ consent.

## 3. Diffusion Tensor Imaging

Diffusion tensor imaging data were acquired at 14 months after the onset of head trauma using a 1.5T with 32 non-collinear diffusion sensitizing gradients by single-shot echo-planar imaging. For each of the 32 non-collinear diffusion sensitizing gradients, 67 contiguous slices were acquired parallel to the anterior commissure-posterior commissure line. Imaging parameters were as follows: acquisition matrix = 96 × 96; reconstructed matrix = 128 × 128; field of view = 221 × 221 mm^2^; TR = 10,726 ms; TE = 76 ms; parallel imaging reduction factor (SENSE factor) = 2; EPI factor = 49; b = 1000 s/mm^2^; NEX = 1; a slice thickness of 2.3 mm with no gap (acquired voxel size 1.25 × 1.25 × 2.5 mm^3^). The Oxford Centre for Functional Magnetic Resonance Imaging of the Brain (FMRIB) Software Library was used for analysis of DTI data. Affine multi-scale two-dimensional registration was used for correction of head motion effect and image distortion due to eddy current. FMRIB Diffusion Software with routines option (0.5 mm step lengths, 5000 streamline samples, and curvature thresholds = 0.2) was used for fiber tracking [17]. For reconstruction of the CST, the seed ROI was placed on the lower pons (anterior blue portion on the color map) and the target ROI was placed on the primary motor cortex (anterior boundary: precentral sulcus; posterior boundary: central sulcus; medial boundary: the midline between the right and left hemispheres; lateral boundary: the line passing through the lateral margin of the precentral knob and horizontal to the midline). For the analysis of the CFTs from the dorsal PMC (dPMC-CFT) and SMA (SMA-CFT), the seed region of interest (ROI) was placed on the crus cerebri on the FA map. The target ROIs were placed on the dPMC (anterior boundary: the line joining the anterior extent of the SMA; posterior boundary: precentral sulcus; medial boundary: the lateral margin of the SMA; lateral boundary: the line passing through the lateral margin of the precentral knob and horizontal to the midline), and the SMA (anterior boundary: the line drawn through the anterior commissure perpendicular to the anterior/posterior commissure line; posterior boundary: anterior margin of M1; medial boundary: midline between the right and left hemispheres; lateral boundary: the line 10.6% lateral from the midline, the ratio of 15 mm to the maximum width of the Montreal Neurological Institute atlas) [18,19]. The fractional anisotropy (FA) and mean diffusivity (MD) values, as well as the tract volume (TV) of the CST, SMA-CFT, and dPMC-CFT were obtained in both hemispheres. Statistical analyses were performed using SPSS software (v. 25.0; SPSS, Chicago, IL, USA). We performed analysis using Bayesian statistics for the determination of differences in FA, MD, and TV of the patient and the control group [20].

On 14-month DTT configuration, the integrities of the left CST, SMA-CFT, and dPMC-CFT were well-preserved (Figure 1B). The results of the Bayesian statistical analyses comparing DTT parameters of the patient and control group are summarized in Table 1. Significant differences were not observed for the FA, MD, and TV values of the CST, SMA-CFT, and dPMC-CFT in both hemispheres between the patient and the control group (*p* > 0.05).

## 4. Transcranial Magnetic Stimulation

TMS was also performed at 14 months after onset using a Magstim Novametrix 200 magnetic stimulator with a 9 cm mean diameter circular coil (Novametrix Inc.Wallingford, CT, USA). Cortical stimulation was performed with the coil held tangentially over the vertex. The left hemisphere was stimulated by a counterclockwise current, and the right hemisphere was stimulated by a clockwise current. Motor-evoked potentials (MEPs) were obtained from both abductor pollicis brevis muscles in a relaxed state. The excitatory threshold (ET) was defined as the minimum stimulus required to elicit an MEP with a peak-to-peak amplitude of 50 μV or greater in two out of four attempts. Stimulation intensity was set at the ET plus 20% of the maximum stimulator output. On a 14-month TMS study, MEPs were obtained at the right abductor pollicis brevis muscle during stimulation of the left hemisphere with 60% of maximal output (the MEP of shortest latency; 21.0 msec, amplitude: 5.1 mV) (Figure 1C).

## 5. Discussion

In this study, we compared the changes of the CST on DTT and TMS, and the CFTs from the secondary motor area on DTT in a patient with mild TBI who showed severe weakness of her right arm. Using electromyography study, we could rule out the possibility of peripheral neuropathy. The contralateral CST is mandatory for the execution of the finger extensor, for which this patient showed nearly complete weakness (1/5), therefore, the precise evaluation of the state of the CST was mandatory in order to rule out the possibility of injury of the contralateral CST [4,5,21]. In addition, the state of the CFTs from the secondary motor area should also be evaluated in order to rule out the possibility of limb-kinetic apraxia [6,22,23,24].

There were no significant differences in DTT parameters (FA, MD, and TV) of the CST, SMA-CFT, and dPMC-CFT between the patient and control group. The FA value represents the state of white matter organization by indicating the degree of directionality while the MD value indicates the magnitude of water diffusion [5,8]. The TV value indicates the number of voxels included in a neural tract, thereby suggesting the total number of fibers within that tract [5,8]. Therefore, no difference of 14-month DTT parameters (FA, MD, and TV) of the CST, SMA-CFT, and dPMC-CFT in the patient compared with those of the control group indicates no abnormality of these neural tracts in this patient. The configurational analysis of the CST, SMA-CFT, and dPMC-CFT on the 14-month DTT also showed well-preserved configuration. In addition, the latency of MEP, which indicates the fastest velocity of a neural tract, can discriminate whether the MEP originated from the CST and the amplitude of MEP suggests that the total fiber numbers of the CST were within normal range in the 14-month TMS study [9,25]. As a result, we could rule out the possibility of the injury of the contralateral CST and limb-kinetic apraxia due to injury of the contralateral CFTs from the secondary motor area.

In conclusion, conversion disorder was confirmed in a patient with mild TBI, who showed severe weakness of an arm, using DTT and TMS. Our results suggest the usefulness of an evaluation of the CST and CFTs from the secondary motor areas using DTT, and the CST using TMS for patients who complain of motor weakness due to conversion disorder. To our best knowledge, this is the first study to demonstrate conversion disorder in patients with motor weakness, using DTT and TMS. However, this study is limited to a case report. Further complementary studies involving larger case numbers are warranted. In addition, DTT is a powerful anatomic imaging tool, which can demonstrate gross fiber architecture; however, due to crossing fiber or partial volume effect, DTT can produce both false positive and negative results throughout the white matter of the brain [26,27].

## Figures and Tables

**Table 1:** Results of the Bayesian statistics analyses of diffusion tensor tractography (DTT) parameters of the left corticospinal tract (CST) and corticofugal tracts (CFT) of the patient and the control group.

		Diffusion Tensor Tractography		
		Patient	Control	*p*-Value
CST	FA	0.47	0.49	0.58
	MD	0.81	0.83	0.57
	TV	1695	1780.25	0.50
SMA-CFT	FA	0.37	0.39	0.60
	MD	1.01	0.99	0.75
	TV	6111	6329.25	0.87
dPMC-CFT	FA	0.35	0.37	0.55
	MD	1.08	1.01	0.37
	TV	6130	6537.5	0.79

DTT: diffusion tensor tractography, CST: corticospinal tract, SMA: supplementary motor area, CFT: corticofugal tract, dPMC: dorsal premotor cortex, FA: fractional anisotropy, MD: mean diffusivity, TV: track volume.
